# Influence of immediate versus delayed dentin sealing on marginal integrity in indirect composite restorations

**DOI:** 10.6026/973206300222546

**Published:** 2026-04-30

**Authors:** Parthivi Singh, Kirti Rathee, Anju S. Hussain, M. Srisha Shivangi, Pragya Patel, Khushboo Magwa

**Affiliations:** 1 Department of Conservative Dentistry and Endodontics, Peoples Dental Academy, Peoples University, Bhopal, Madhya Pradesh, India; 2 Department of Conservative Dentistry and Endodontics, Institute of Dental Studies and Technologies, Ghaziabad, Uttar Pradesh, India; 3 Department of Conservative Dentistry and Endodontics, District Hospital, Anuppur, Madhya Pradesh, India; 4 Department of Conservative Dentistry and Endodontics, Modern Dental College and Research Centre, Indore, Madhya Pradesh, India; 5 Department of Conservative Dentistry and Endodontics, People's College of Dental Sciences and Research Centre, Peoples University, Bhopal, Madhya Pradesh, India

**Keywords:** Immediate dentin sealing (IDS), delayed dentin sealing (DDS), marginal integrity, indirect composite restoration, microleakage

## Abstract

Marginal integrity of indirect composite restorations is critical for clinical longevity and the timing of dentin sealing may influence the tooth-restoration interface. Therefore, it is of interest to evaluate the effect of immediate versus delayed dentin sealing on marginal integrity of indirect composite restorations. Sixty extracted premolars with standardized cavities were divided into three groups (IDS, DDS control), restored with indirect composite inlays and assessed after thermocycling using stereomicroscopy and SEM. The IDS group demonstrated significantly smaller marginal gaps and lower microleakage compared to DDS and control groups (p<0.001). Immediate dentin sealing provides superior marginal adaptation and may enhance the clinical performance of indirect composite restorations.

## Background:

The indirect composite restorations have been very popular in modern day restorative dentistry because of their good esthetic qualities, improved mechanical properties through additional oral polymers and capacity to give better proximal contacts and anatomical shapes than the direct restorations [1]. These restorations such as inlays, onlays and overlays are specified in moderate to large cavities where direct composite placement can be affected by polymerization stresses of shrinkage and problems of obtaining the best contour [2]. Although adhesive technology and composite material have improved technology, marginal integrity of the indirect restorations is among the most important factors that determine the success of these restorations in the long run clinical use [3]. Marginal integrity is defined as the quality and integrity of interface existing between the restoration margin and the prepared tooth structure. Weaknesses at this interface express themselves in the form of marginal gaps, microleakage and finally marginal discoloration, secondary caries and restoration failure [4]. The resin cement layer between the restoration and the tooth is the inherently weakest part of the adhesive assembly and the quality of the cementation directly affects the bonding protocol used during the cementation phase [5]. Immediate dentin sealing was a concept proposed to have as a solution to various drawbacks of the delay theory of bonding that existed during cementation [6]. In the IDS method, an agent of dentin bonding is applied on clean dentin immediately after preparing the tooth and before taking an impression. The strategy takes advantage of the best bonding environments found on the freshly prepared dentin, which contains no elements of provisional cement, saliva, or bacterial biofilm [7]. A number of studies have shown that IDS gives dentin value of higher bond strength values than delayed dentin sealing where the adhesive is applied during definitive cementation [8]. The argument behind the use of IDS is not restricted to the strength of bonds. New dentin is in a good condition of being hybridized due to the presence of a smear layer and collagen network. Application of an adhesive system leads to the formation of a resin-filled hybrid layer that will be used as an effective seal of the dentinal tubules and will reduce postoperative pain and protect the pulp-dentin complex during the provisional period [9]. In addition, the sealed surface of dentin offers a resin to resin bonding substrate during cementation which has been demonstrated to be more predictable and reliable than bonding the resin cement to dentin directly [10]. However, the delayed dentin sealing method consists of applying the adhesive system during the cementation, when the provisional restoration was removed.

This protocol exposes the ready dentin to possible pollution by interim cement substances that can negatively influence the standard of the hybrid layer and the bond integrity [11]. Remaining non-temporary cement elements especially those containing the eugenol have been found to hinder the polymerization of resin-based materials and weaken the strength of adhesive bonds [12]. Although many studies have compared bond strength benefits of IDS compared to DDS, not as many studies have directly compared the effect of these sealing protocols on the marginal integrity of indirect composite restorations when subjected to simulated clinical aging environments [13]. The available literature has some contradictory findings on the extent of benefit that is transferred by IDS to marginal adaptation, especially when the use of modern universal adhesive systems and eugenol-free temporary cements are in use [14]. Moreover, the majority of the past researches have been directed at ceramic restorations and no available data specifically on indirect composite restorations are complete [15]. Therefore, it is of interest to test and compare the effects of the instant dentin sealing versus the delayed dentin sealing on the marginal structure of indirect compounds restorations, which was measured by means of the marginal gap measurement, microleakage testing and scanning electron microscopic analysis.

## Materials and Methods:

An experimental design was adopted in the study, with the sample being prepared prior to the research. This experimental study was done *in vitro* and at the dental research laboratory after receiving an institutional ethical permission to use extracted human teeth. A total of 60 freshly extracted human maxillary premolars (extracted due to orthodontic reasons) were opted and immersed in 0.1% thymol solution at 4°C not exceeding three months before they were used. The teeth were inspected at 4 x magnifications to rule out those that had cracks, caries, prior restorations or developmental defects.

## Inclusion criteria:

Intact human maxillary premolars of similar sizes (buccolingual width 8.510 mm), no structural defects, extracted in the three months.

## Exclusion:

Teeth with apparent cracks, carious lesions, hypoplastic defects, prior restorative or endodontic therapy or root fractures.

## Tooth preparation:

All the teeth were fixed on self-curing acrylic resin blocks as low as 2 mm beneath the cementoenamel junction to mimic the alveolar bone level. A single trained operator was used to prepare standardized mesio-occluso-distal (MOD) cavity preparations with high-speed handpiece with abundant water cooling and cylindrical diamond burs (ISO #835, medium grit). The standardization of the preparation dimensions were as follows; 4 mm buccolingual width, 2 mm pulpal floor depth starting at the central fossa and proximal box 1 mm above the cementoenamel junction; and gingival seat width of 1.5 mm. Internal angles were rounded and the walls of the cavities had a divergence of 6 degrees to the occlusal surface. Diamond burs were changed after 5 preparations to ensure efficiency in cutting.

## Group allocation:

The ready teeth were organized randomly as specimen groups of 20 members each with the help of a computer-generated randomization table:

[1] Group 1 (IDS group, n=20): The dentin was sealed immediately after cavity preparation, before taking the impression.

[2] Group 2 (DDS group, n=20): The dentin sealing was not performed until definitive cementation.

[3] Group 3 (Control group, n=20): No dentin sealing was done; resin cement was applied directly onto unprepared dentin at cementation.

## Immediate dentin sealing protocol (Group 1):

In the IDS group, the cavity was sprayed with water and air-dried softly after tooth preparation to ensure that the surface of the dentin was moist. A two-step self-etch adhesive system (Clearfil SE Bond 2, Kuraray Noritake, Tokyo, Japan) was then applied as per the instructions of the company. The self-etch primer was placed on the dentin surfaces 20 seconds with active agitation followed by 5 seconds of air drying. A uniform, thin layer of the bonding resin was then coated and cured under 20 seconds of irradiation by an LED curing unit (Elipar DeepCure-S, 3M, St. Paul, MN, USA) with an irradiance of 1, 470 mW/cm_2_. Glycerin gel was applied thinly and another 10-second light cure was done to make sure that the oxygen-inhibited layer was fully polymerized. Unused adhesive was eliminated at the margins of the enamels with a fine-grit diamond bur under magnification.

## Impression and provisional restoration:

All preparations were made and impressions obtained of the preparations using the dual-arch technique with Polyvinyl siloxane impressions (Aquasil Ultra+, Dentsply Sirona, Charlotte, NC, USA). Temporary resins were made out of a bis-acryl composite provisional material (Protemp 4, 3M) and bonded with eugenol-free temporary cement (TempBond NE, Kerr, Orange, CA, USA). Each of the specimens was kept in distilled water at 37°C/14 days to represent the clinical provisional period. Fabrication of indirect composite inlays involves a reduced number of procedures that are required to be performed. Fabrication of indirect composite Inlays- This is where fewer steps need to be taken to create inlays. Type IV dental stone was used to cast the working casts (Fujirock EP, GC Corporation, Tokyo, Japan). The indirect composite inlays were made in small steps of 2-mm increments with light-polymerized increments of 40 seconds in a laboratory composite resin system (Gradia Indirect, GC Corporation). The end polymerization took place in a laboratory light-curing unit (Labolight DUO, GC Corporation) by a 5-minute polymerization. The inside of all inlays was air-brushed using aluminum oxide particles at 2-bar pressure over 10 seconds and ultrasonically rinsed using ethanol in 3 minutes.

## Cementation procedures:

Temporary restorations were then meticulously stripped away and the preparations washed with pumice slurry and water using a rubber cup at low speed. Leftover transient cement was carefully eliminated.

## Group 1 (IDS):

The bonded surface was brushed with aluminum oxide in air at 1-bar during 5 seconds to air-cleanse the surface. The inlay was air-dried and left to dry on the internal surface with a silane coupling agent (Monobond Plus, Ivoclar Vivadent, Schaan, Liechtenstein) applied on the inlay surface. The inlay was coated using a dual cure resin cement (Variolink Esthetic DC, Ivoclar Vivadent) and firmly seated using a finger pressure. Unused cement was eliminated and light curing was done on both surfaces (occlusal, buccal, lingual) 40 seconds.

## Group 2 (DDS):

This group used an adhesive system (Clearfil SE Bond 2) on the dentin during cementation, as in the case of IDS. The cement was allowed to cure to 20 seconds before cementation took place. The next step was then the same as Group 1, cementation.

## Group 3 (Control):

Adhesive was not placed on dentin. The cement in resin was applied as per the instructions of the manufacturer. The cementation was done in a similar manner.

## Thermocycling:

All cemented samples were left in distilled water at 37°C temperature and then thermocycler 10,000 times at 5°C -55°C water baths, dwell time used 30 s and transfer time used 10s.

## Marginal gap measurement:

The specimens were embedded in epoxy resin and mesiodistally sectioned by the center of the restoration with a slow-speed diamond saw (Isomet 1000, Buehler, Lake Bluff, IL, USA) in water. Polishing of the sectioned surfaces was done in a succession using a 600-, 1200- and 2000-grit silicon carbide paper. The four pre-determined points (two occlusal margins and two gingival margins) per section were measured at 40 x with a stereomicroscope (SZX16, Olympus, Tokyo, Japan) and image analysis software (ImageJ, NIH, Bethesda, MD, USA). At every location three measures were made and the average was calculated.

## Microleakage assessment:

Two layers of nail varnish were applied on separate samples of each group (n=10) and a 1-mm window was left around the restoration margins. The specimens were dipped in 2% solution of methylene blue dye and kept at 37°C in 24 hours, rinsed thoroughly and cut into sections as mentioned above. The penetration of dye was measured using the stereomicroscope and rated on a 4-point ordinal scale, 0 = no dye penetration; 1 = dye penetration up to a third of the cavity wall; 2 = dye penetration up to a third of the cavity wall; 3 = dye penetration up to the entire cavity wall to the pulpal floor. The methodology that was used to support the results is Scanning Electron Microscopy. SEM analysis of representative samples of each group (n=3) was done. Dehydration of sections in sequential ethanol solutions, sputter-plating with gold-palladium and observation under a field-emission scanning electron microscope (JSM-7600F, JEOL, Tokyo, Japan) at accelerating voltages of 10 -15 kV were carried out. The interfaces of the tooth-cement-restoration were tested at the magnification of 100x to 5000x.

## Statistical analysis:

The analysis of data was done in SPSS version 28.0 (IBM Corp., Armonk, NY, USA). Shapiro-Wilk test was used to test normality. Marginal gap data were compared by means of one-way ANOVA with post hoc test of Tukey which was the comparison of the two means. The scores of the microleakage were tested with Kruskal-Wallis test and Dunn post hoc test with Bonferonni correction. The probability value was set to 0.05.

## Results:

The mean marginal gap width showed statistically significant differences among the three groups (p < 0.001). The IDS group demonstrated the smallest marginal gaps at both occlusal and gingival margins, whereas the control group exhibited the largest values, with the DDS group showing intermediate results. Pairwise comparisons confirmed significant differences between all groups. Additionally, gingival margins consistently presented higher marginal gap values than occlusal margins across all groups (Table 1 - see PDF). Across all groups, gingival margins exhibited significantly larger gaps compared to occlusal margins (p < 0.01). The distribution of microleakage scores differed significantly among the three groups (H = 31.46, p < 0.001). The IDS group demonstrated the lowest microleakage scores, with the majority of specimens showing no dye penetration or minimal penetration. The control group exhibited the highest microleakage scores, with several specimens showing dye penetration extending to the pulpal floor. The results are presented in Table 2 (see PDF). A two-way analysis was conducted to examine the interaction between sealing protocol and margin location. Both factors significantly influenced marginal gap values and no significant interaction was detected. The comparative data are presented in Table 3 (see PDF). SEM examination revealed distinct differences in the interfacial morphology among the three groups. In the IDS group, a continuous, well-defined hybrid layer was observed at the dentin-adhesive interface, with complete resin tag penetration into dentinal tubules. The cement-adhesive interface appeared intimate and void-free. In the DDS group, the hybrid layer appeared less uniform, with occasional gaps and incomplete resin tag formation. The control group exhibited the most significant interfacial deficiencies, with evident gaps between the cement and dentin, irregular resin penetration and occasional areas of complete debonding at the cervical margins.

## Discussion:

The findings of the current research proved that the immediate dentin sealing was of great help in enhancing the marginal integrity of the indirect composite restorations in relation to delayed dentin sealing and the no sealing control protocol. As a result, the null hypothesis that there would be no significant difference between marginal integrity in sealing protocols was rejected. The mechanisms that can explain the superior marginal integrity of the IDS group are connected and can be explained in several ways. The use of an adhesive system to newly cut dentin provides the best environment to form hybrid layers. Recently polished dentin layer has a smear layer that is uniform and the collagen fibrils are patent which is very receptive to resin infiltration [16]. The hybrid layer which grows under such ideal situations is more complete and denser than one which grows over the dentin which has been exposed to the oral environment or is contaminated with provisional materials [17]. The adhesive layer of the IDS protocol is polymerized and stress relaxation of adhesive layer occurs in the provisional stage and it has been suggested as one of the factors that enhance the bond performance. Since the adhesive would be light-cured without the restoration, polymerization contraction stresses would be exerted upon the prepared tooth surfaces, which facilitates close adherence of the adhesive to dentin. Conversely, with bonding carried out concomitantly with cementation using the DDS procedure, the opposing contraction stresses of the adhesive and cement can jeopardize interfacial integrity [18]. These relatively large marginal gaps of the DDS group relative to the IDS group is as expected by results in past studies who have established the harmful impacts of provisional cement contamination on dentin bonding. Although eugenol-free temporal cement is used in the current research, remnants of cement substances may block dentinal tubules and change the surface chemistry of prepared dentin, which may inhibit adhesive penetration and compromise hybrid layer quality [19]. Pumice and water cleaning protocols, although the standard clinical practice, can fail to fully remove all the contaminants to the dentin surface [20]. The observation that gingival margins had very large gaps as compared to occlusal margins in all groups is clinically significant and is consistent with the past reports. Various factors have been attributed to this phenomenon such as the closeness of the gingival margin to the cementoenamel junction whereby there is a reduction in enamel thickness, ability to deliver sufficient polymerization of the resin cement at the deeper position of the gingival and ensuring the full seating of the restoration at the floor of the proximal box [21]. The IDS protocol also seemed to correct this issue to a degree with IDS group showing a similar or smaller value of gingival gap than the value of occlusal gap in other groups. The findings of the microleakage were similar to the marginal gaps and again indicated the better effect of the IDS approach. The unusable dentin in the IDS group serves as a blockage of the flow of fluids via the dentinal tubules, which practically removes one of the main avenues of microleakage at the tooth-restoration interface [22]. The clinical significance of the finding is supported by the high percentage of score 0 samples in the IDS group (70) relative to those of the DDS (30) and control (10) groups. The secondary caries formation has been directly attributed to marginal microleakage and is the most frequent cause of indirect restoration replacement [23].

The quantitative findings were corroborated by the qualitative information that was obtained through the SEM observations. The well- defined continuous hybrid layer that is evident in the IDS group is cases of optimal resin-dentin inter diffusion, which forms the basis of stable adhesive bonding. The unstable and jagged hybrid layer observed in both the DDS and control indicates inefficient resin infiltration and would form weak spots that are liable to hydrolytic damage and mechanical breakdown in the long run [24]. In addition, the resin-resin bonding interface formed in the IDS protocol where the resin cement is bonded to a pre-polymerized adhesive layer and not to dentin itself is also far more predictable and technique-insensitive [25]. The thermocycling regime used in the present study (10, 000 cycles) represents a period of about one year of clinical service and presents thermal stresses posing difficulties to the interfacial integrity of the restorations. The retained superiority of the IDS group following the thermocycling implies that bonding attained using this protocol is less thermal degraded than the DDS and control protocols [26]. The implications of this finding to the clinical prognosis of long-term restorations of the indirect composite restorations are significant. It is also worth mentioning that the application of self-etch adhesive system in this paper will allow avoiding the phosphoric acid etching and make the IDS procedure less complicated and minimize the chances of over-etching or post-operative sensitivity. Self-etch adhesives form a layer that combines both smear layer and hybrid layer, which causes a gradual transition zone which is potentially more resistant to nanoleakage than the hybrid layer created by etch-and-rinse adhesives [27]. Nevertheless, the adhesive system can also determine the comparative advantage of IDS and more research is justified on the comparisons of the adhesive categories to IDS and DDS. The practical use of these findings in clinical practice is that IDS should be used when indirect composite restorations are planned. The intervening clinical procedure of using an adhesive as soon as it is prepared offers only a limited amount of chair time (about 23 minutes) but has significant advantages in terms of marginal integrity, minimized microleakage and possible prolonged restoration life [28]. The sealed dentin also offers a protective layer in the provisional period, which lowers the chances of bacterial invasion of the pulp-dentin complex and limits the post-operative sensitivity [29]. There are a few weaknesses of this study that ought to be mentioned. Although *in vitro* design enables the creation of the controlled experimental conditions, it cannot be entirely replaced with a real oral environment, featuring masticatory loading, enzymatic degradation and bacterial biofilm formation. Thermocycling protocol, which is a popular one, is merely a thermal aging and it does not include mechanical fatigue. There are chances that the extracted teeth could add some variability in dentin quality and permeability potentially affecting the outcomes of bonding [30]. These *in vitro* findings need to be confirmed by long-term clinical studies in order to come up with concrete clinical recommendations. In addition, this research paper only tested a single adhesive system and single resin cement and the findings cannot be extrapolated to other material combinations. Additional studies are needed to determine the impact of alternative adhesive plans (universal adhesives in alternative modes), alternative resin cement formulations and other indirect restorative substances to the IDS-marginal integrity association [31]. Achieving optimal marginal adaptation and minimizing microleakage are essential for the long-term success of indirect restorations [32].

## Conclusion:

The timing of dentin sealing plays a significant role in determining the marginal integrity of indirect composite restorations. Immediate dentin sealing enhances interfacial adaptation and reduces marginal discrepancies compared to delayed approaches. Incorporating appropriate sealing protocols can improve restoration performance and long-term clinical outcomes.
